# Supplementary material for: Improving effect size estimation and statistical power with multi-echo fMRI and its impact on understanding the neural systems supporting mentalizing
Source: Neuroimage. 2016 Nov 15;142:55–66. doi: 10.1016/j.neuroimage.2016.07.022 (PMC5102698; doi:10.1016/j.neuroimage.2016.07.022)
Supplement: Supplementary Table 1 — Classes of signal sources decomposed by ME-ICA. Signal sources elucidated through combination of multivariate decomposition (PCA, ICA in order) and T2* decay analysis of multi-echo fMRI data as implemented in ME-ICA. κ is pseudo-F statistic component-level TE-dependent scaling suggesting network BOLD origin. ρ is pseudo-F statistic component-level TE-independent scaling suggesting artifact. [file mmc1.docx]

|  | ***%*Δ*S vs. TE*** | ***Model significance*** | ***Distribution*** | ***Decom-position***  ***source*** | ***Contrast Mechanism*** | ***Interpretation*** |
| --- | --- | --- | --- | --- | --- | --- |
| 1 | Linear scaling (1% @ TE=10ms, 2% @ TE=20ms, etc) | Significant (κ🡺p<0.025):  TE-dependence | Sparse/in-dependent | ICA | T_2_* only | BOLD Functional Network |
| 2 | Constant (1% @ TE=10ms, 1% @ TE=20ms, etc) | Significant  (ρ🡺p<0.025):  TE-independence | Sparse/in-dependent | ICA | S_0_ only (motion, flow, gradient) | Non-BOLD Artifact |
| 3 | Neither (1) or (2) | Low significance  (κ,ρ🡺p>>0.1) | Gaussian | PCA | Thermal | Random noise |
| 4 | Both (1) or (2) | Mixed moderate  significance  (κ,ρ🡺p<0.1) | Sparse/in-dependent | ICA | T_2_* & Flow | Venous drainage |
| 5 | Both (1) or (2) | Mixed moderate significance  (κ,ρ🡺p<0.1) | Gaussian | PCA | Mixed | Incomplete decomposition |

**Supplementary Table 1: Classes of signal sources decomposed by ME-ICA.** Signal sources elucidated through combination of multivariate decomposition (PCA, ICA in standard sequence) and T2* decay analysis of multi-echo fMRI data as implemented in ME-ICA. κ is pseudo-F statistic component-level TE-dependent scaling. ρ is pseudo-F statistic component-level TE-independent scaling.
